# Impact of Body Mass Index on the Outcomes of Catheter Ablation of Atrial Fibrillation: A European Observational Multicenter Study

**DOI:** 10.1161/JAHA.119.012253

**Published:** 2019-10-04

**Authors:** Rui Providência, Pedro Adragão, Carlo de Asmundis, Julian Chun, Gianbattista Chierchia, Pascal Defaye, Frédéric Anselme, Antonio Creta, Pier D. Lambiase, Boris Schmidt, Shaojie Chen, Diogo Cavaco, Ross J. Hunter, João Carmo, Stephane Combes, Shohreh Honarbakhsh, Nicolas Combes, Maria João Sousa, Zeynab Jebberi, Jean‐Paul Albenque, Serge Boveda

**Affiliations:** ^1^ Clinic Pasteur of Toulouse Toulouse France; ^2^ St. Bartholomew's Hospital Barts Health NHS Trust London United Kingdom; ^3^ Institute of Health Informatics Research University College of London United Kingdom; ^4^ Cardiology Department Hospital de Santa Cruz Lisbon Portugal; ^5^ Heart Rhythm Management Centre Universiteit Ziekenhuis Brussel Belgium; ^6^ Postgraduate program in Cardiac Electrophysiology and Pacing Vrije Universiteit Brussel Belgium; ^7^ Cardioangiologisches Centrum Bethanien Medizinische Klinik III, Markus Krankenhaus Frankfurt am Main Germany; ^8^ CHU Michallon Grenoble France; ^9^ University Hospital Rouen France

**Keywords:** atrial fibrillation, metabolic syndrome, obesity, sinus rhythm, vascular complications, Atrial Fibrillation, Catheter Ablation and Implantable Cardioverter-Defibrillator

## Abstract

**Background:**

Outcomes of catheter ablation of atrial fibrillation (AF) are variable and the predictors of success require further elucidation since the identification of correctable risk factors could help to optimize therapy. We aimed to assess the impact of body mass index (BMI) in the overall safety and efficacy of catheter ablation of AF, with emphasis on the use of cryoballoon ablation and novel oral anticoagulants.

**Methods and Results:**

There were 2497 consecutive patients undergoing catheter ablation of AF in 7 European high volume centers were stratified according to BMI (normal weight <25 kg/m^2^, pre‐obese 25–30 kg/m^2^, obesity 30–35 kg/m^2^, and morbid obesity ≥35 kg/m^2^) and comparisons of procedural outcomes evaluated. Pre‐obese and obese patients presented more comorbidities (hypertension, diabetes mellitus, and sleep apnea), and had higher rates of non‐paroxysmal AF ablation procedures. The rate of atrial 12‐month arrhythmia relapse increased alongside with BMI (35.2%, 35.7%, 43.6%, and 48.0% *P*<0.001). During a median follow‐up of 18.8 months (interquartile range 11–28), after adjusting for all baseline differences, BMI was an independent predictor of relapse (hazard ratio=1.01 per kg/m^2^; 95% CI 1.01–1.02; *P*=0.002), adding incremental predictive value to obstructive sleep apnea. BMI was not a predictor for any of the reported complications. Using novel oral anticoagulants and cryoballoon ablation was safe and efficacy was comparable with vitamin‐K antagonists and radiofrequency ablation.

**Conclusions:**

Obese patients present with a more adverse comorbidity profile, more advanced forms of AF, and have lower chances of being free from AF relapse after ablation. Use of novel oral anticoagulants and cryoballoon ablation may be an option in this patient group.

**Clinical Perspective**

**What Is New?**

- We observed a comparable performance and safety of cryoballoon ablation, and novel oral anticoagulants was observed for patients with high body mass index.
- Procedural safety was comparable for this population.
- Freedom from atrial arrhythmia relapse after blanking was lower for obese patients, even after adjustment for obstructive sleep apnea and other potential confounders.

**What Are the Clinical Implications?**

- Cryoballoon ablation and using novel oral anticoagulants may be alternatives for the treatment of patients with high body mass index referred to catheter ablation of atrial fibrillation.

## Itroduction

Catheter ablation of atrial fibrillation (AF) has emerged as an effective treatment option, and now has a Class I indication in symptomatic patients with drug‐refractory AF.^1^, ^2^ The success rate of this procedure, and consequently freedom from AF or atrial tachycardia relapse, is >70% at 12 months for paroxysmal AF,^3^ and close to 50% at 18 months for persistent AF.^4^

Addressing correctable causes of AF relapse is of interest, as this may allow improvement of the outcomes of this procedure. Preliminary evidence from small studies suggested that obese patients present higher relapse rates.^5^, ^6^ However, a meta‐analysis has shown that this could happen as a result of associated comorbidities in overweight patients and not body mass index (BMI) itself.^7^

Data on the performance of noval oral anticoagulants (NOACs) in the setting of catheter ablation and cryoballoon ablation in obese patients are scarce.^8^, ^9^, ^10^

We aimed to clarify this matter and assess the impact of BMI on outcomes of AF ablation, adjusting for associated comorbidities.

## Methods

The data that support the findings of this study are available from the corresponding author upon reasonable request and approval for the principal investigator from each participating center.

### Setting and Patient Population

This is a non‐randomized observational study conducted at 7 European centers. We compared procedural and mid‐term outcomes of patients who underwent catheter ablation of AF, based on World Health Organization BMI categories (normal weight: 18.5–25 kg/m^2^; pre‐obesity: 25–30 kg/m^2^; obesity: 30–30.5 kg/m^2^; and morbid obesity: ≥35 kg/m^2^)^11^ and then assessed BMI as a potential independent predictor of AF/atrial tachycardia relapse.

Among all patients aged >18 years undergoing a left atrial ablation procedure during a 24 months’ time interval, the eligibility criterion was presence of AF refractory to at least one Class I or Class III anti‐arrhythmic drug. All patients provided written informed consent before the procedure. No patients were excluded from the study as a result of acute complications. The study complied with the 2013 Declaration of Helsinki and the research protocol was approved by the local ethics committees. Participants provided their informed consent for an ablation procedure and for data to be used for research purposes.

Centers contributing to this paper were asked to provide data of all consecutive patients ablated during a minimum of 6 months, during 2014 and 2015. At that time, the following annual AF ablation volumes were observed: Toulouse 700, Frankfurt 700, Brussels 500, London 350, Lisbon 250, Grenoble 200, and Rouen 150 AF ablations‐per‐year.

### Pre‐Procedural Assessment

All variables at the time of the procedure were defined and categorized according to the literature or common practice. Information was collected on demographics, admission day anthropometric data, clinical comorbidities based on patients’ notes and referral letters. Patients with a history suggestive of obstructive sleep apnea (OSA) were routinely referred for screening by local sleep specialists. Data from the referral transthoracic echocardiogram were analyzed, and a multislice computed tomography scan imaging of the left atrium was systematically collected.

### Procedural Details on Ablation Procedures

Procedures were performed under sedation or general anesthesia, according to each institution's protocol. Venous access was obtained via the femoral vein. A quadripolar or decapolar catheter was positioned in the coronary sinus in all patients as a reference and for pacing. In the absence of patent *foramen ovale*, a single or dual transseptal puncture was performed under fluoroscopic guidance. Transesophageal echocardiography was used based on operator preference. Patients received intravenous heparin to maintain an activated clotting time of 300 to 350 seconds upon completion or before the transseptal puncture, according to each institution's protocol. The transseptal sheaths throughout were continuously flushed with heparinized saline.

Details of the AF ablation technique and peri‐procedural management at our institutions have been published previously.^12^, ^13^, ^14^, ^15^ Basically, pulmonary vein isolation was the main procedural end point, and was performed as a first step in all procedures. If the patient was in AF at the start of the procedure and the arrhythmia organized into an atrial tachycardia, this was mapped and ablated. In patients undergoing cryoballoon ablation, or other techniques different from standard point‐by‐point radiofrequency ablation, if the patient remained in AF after isolation of all 4 pulmonary veins, direct‐current cardioversion to sinus rhythm was performed and no further ablation undertaken. Electroanatomic mapping was not used in any of the cryoablation cases. In patients undergoing radiofrequency ablation of persistent AF and not cardioverting to sinus rhythm or not organizing to atrial tachycardia during ablation we mapped and ablated areas of complex fractionated atrial electrograms in both atria and the coronary sinus and subsequently DC cardioverted the patient if AF persisted. If patients organized into atrial tachycardia while having their ablation performed, the tachycardia was mapped and ablated.

### Follow‐Up

A systematic transthoracic echocardiography was performed before discharge. Patients were also evaluated at 3, 6, and 12 months after the procedure. Information collected during follow‐up included a 12‐lead ECG and 24‐hour Holter monitoring at each visit. Additional patient visits and further testing were allowed in case of symptoms. After the first year, follow‐up was performed on an annual basis. Antiarrhythmic drugs were prescribed at discharge only for specific indications (ie, relapse during the admission, need for cardioversion, longstanding persistent AF, etc) and at the operator's discretion. In those instances, antiarrhythmic drugs were stopped after the first 3 months in the absence of recurrence. The first 3 months post‐procedure were considered blanking period.

### End Points and Safety Concerns

Recurrence was defined as any symptomatic or asymptomatic atrial arrhythmia lasting >30 seconds following the 3 months blanking period after catheter ablation. Patients with relapse during the blanking period with no response to pharmacologic or electrical cardioversion were also classified as having a relapse.

The main efficacy end point was freedom from atrial arrhythmias following a blanking period of 3 months. AF or atrial tachycardia relapse during the initial 3‐month blanking period was also documented.

With regard to safety, information on the following complications was systematically collected: vascular complications (if requiring intervention or prolongation of admission), thromboembolism (transient ischemic attack, stroke and/or systemic embolism happening during or in the first month after the procedure), phrenic nerve palsy post‐procedure, pericardial effusion (if causing haemodynamic instability and/or requiring pericardiocenthesis or prolonged monitoring), esophageal fistula, and procedure‐related death. Other complications were reported at the discretion of the operator.

### Performance of NOACs and Cryoballoon Ablation in Obese Patients

Concern exists about the use of NOACs in obese patients, as using fixed dosing schedules in patients who are above the average weight in the clinical trials may lead to unintentional underdosing, and thus increasing a patient's risk for thrombotic complications. As robust data in this subset of patients are still missing, expert groups have published specific recommendations for the management of anticoagulation in obese patients.^8^, ^9^ The *International Society on Thrombosis and Haemostasis* suggests not using NOACs in patients with BMI >40 kg/m^2^ or weight >120 kg.^8^ The *Anticoagulation Forum* advises that until further evidence is available, NOACs should be avoided in patients >120 kg or BMI ≥35 kg/m^2^ unless vitamin K antagonists cannot be used.^9^ We have therefore performed a sub‐analysis on the efficacy and safety of the NOACs in obese patients. Different cut‐off points were used (BMI >30 kg/m^2^, BMI 35 kg/m^2^, BMI >40 kg/m^2^, and weight >120 kg).

The *FIRE & ICE* trial (a Controlled, Prospective, Non‐Inferiority, Parallel‐Group, Randomised, Interventional, Open, Blinded Outcome Assessment [PROBE‐Design], Multi‐Centre Trial, Comparing Efficacy and Safety of Isolation of the PVs With a Cryoballoon Catheter vs a Radiofrequency Ablation With a ThermoCool Catheter in Patients With PAF) provides the strongest evidence about the comparable efficacy and safety of cryoballoon ablation for AF.^10^ However, even though patients were not excluded based on body weight, this trial included only 762 patients with paroxysmal AF, obese and very obese patients were under‐represented (mean BMI was 28±4.7 kg/m^2^), and no sub‐analysis for different BMI classes were conducted. To address this knowledge gap, we performed a sub‐analysis of patients with a BMI >30 kg/m^2^, comparing the efficacy and safety of cryoballoon ablation versus radiofrequency ablation in paroxysmal and in persistent AF.

### Statistical Analysis

Comparisons were performed across the 3 pre‐specified BMI classes. The Chi‐square was used for ordinal variables (Chi‐square for linearity: linear by linear association) and ANOVA was used for comparison of continuous variables. Levene test was used to check the homogeneity of variance; equivalent non‐parametric tests were used when Kolmogorov–Smirnov was in favor of the absence of normal distribution. Results with *P*<0.05 were regarded as significant.

Kaplan‐Meier curves were traced for illustrating freedom from AF or atrial tachycardia among patients in the different BMI classes, and the log rank *P* test was used for assessing existing differences. Independent predictors of sinus rhythm maintenance after a single ablation procedure were assessed through Cox regression (Method: Forward Likelihood Ration, Probability for Stepwise 0.05; used for Model 1). A subsequent model (Model 2) was created using the previously identified predictors, with additional adjustment for age, sex, LVEF, LA volume, and use of anti‐arrhythmic drugs on discharge.

PASW Statistics version 18.0 was used for descriptive and inferential statistical analysis.

## Results

### Study Population

During the study inclusion period, 2497 patients underwent catheter ablation of AF. No patients were excluded from the study at the time of cohort inception. Only 711 patients had normal weight. The majority of patients were overweight: 1092 were pre‐obese individuals, and 508 were obese, and 186 were morbidly obese.

The majority of patients (70.6%) were men and the mean age was 61.1±10.2 years. Mean CHA_2_DS_2_‐VASc score was 1.6±1.4 and 57.6% of patients had paroxysmal AF. No significant differences were observed in the mean number of procedures each individual received, per BMI class (between 1.2 and 1.3—Table 1). Cryoballoon ablation was used more frequently in the normal BMI group. Additional left atrial ablation beyond pulmonary vein isolation was less frequently performed in the normal BMI group.

**Table 1:** Baseline Characteristics of the Study Population

Variable	Total Sample (n=2497)	Normal Weight (n=711)	Pre‐Obese (n=1092)	Obese (n=508)	Morbidly Obese (n=186)	Overall *P* Value
Age, y	61.1±10.2	61.9±11.2	60.9±9.9	60.7±9.8	60.1±8.8	0.053
Female sex	29.4% (734)	37.0% (263)	22.7% (248)	29.5% (150)	39.2% (73)	<0.001
AF duration, y	5.0±5.4	4.9±5.3	5.2±5.8	4.5±4.4	5.0±5.7	0.129
Paroxysmal AF	57.6% (1438)	66.8% (475)	56.1% (613)	50.8% (258)	49.5% (92)	<0.001
Persistent AF	32.8% (820)	25.9% (184)	34.9% (381)	36.2% (184)	38.2% (71)	
Longstanding persistent AF	3.5% (87)	1.8% (13)	3.1% (34)	6.1% (31)	4.8% (9)	
Left atrial tachycardia	6.1% (152)	5.5% (39)	5.9% (64)	6.9% (35)	7.5% (14)	
Mean N of procedures^a^	1.2±0.5	1.2±0.5	1.2±0.5	1.3±0.5	1.2±0.5	0.206
CHA_2_DS_2_‐VASc	1.6±1.4	1.6±1.4	1.5±1.3	1.7±1.3	2.0±1.4	<0.001
Congestive heart failure	8.0% (199)	7.0% (50)	7.6% (83)	9.4% (48)	9.7% (18)	0.346
Hypertension	45.9% (1146)	33.5% (238)	44.3% (484)	58.1% (295)	69.1% (129)	<0.001
Diabetes mellitus	9.4% (234)	4.8% (34)	7.8% (85)	12.8% (65)	26.9% (50)	<0.001
Stroke or TIA	7.4% (186)	9.0% (64)	7.1% (77)	6.9% (35)	5.4% (10)	0.248
Vascular disease	8.5% (213)	7.3% (52)	9.5% (104)	8.5% (43)	7.5% (14)	0.396
Obstructive Sleep apnea	7.0% (176)	1.4% (10)	6.0% (66)	12.6% (64)	19.4% (36)	<0.001
eGFR, mL/min	75.1±18.4	77.7±18.5	75.3±17.9	72.1±17.9	71.7±20.4	<0.001
Indexed LA volume, mL/m^2^	48.6±18.6	48.1±20.3	48.8±18.1	48.4±17.4	49.9±18.2	0.737
LVEF, %	62±9	63±8	61±9	61±9	61±8	<0.001
LVEF <35%	2.0% (50)	1.1% (8)	1.8% (20)	3.3% (7)	2.7% (5)	0.045
Cryoballoon ablation	29.4% (733)	33.9% (241)	27.6% (301)	26.8% (136)	29.6% (55)	0.015
Use of general anesthesia	67.8% (1692)	68.6% (488)	66.2% (723)	71.5% (363)	63.4% (118)	0.101
Procedure duration, min	135±57	124±49	135±59	142±58	152±64	<0.001
Fluoroscopy duration, min	23±13	23±12	23±13	24±13	25±14	<0.001
CFAE ablation	14.2% (355)	10.0% (71)	13.7% (150)	20.1% (102)	17.2% (32)	<0.001
Linear LA ablation^b^	22.9% (572)	17.9% (127)	21.8% (238)	29.9% (152)	29.6% (55)	<0.001
CTI ablation	21.9% (546)	23.1% (164)	21.1% (230)	22.6% (115)	19.9% (37)	0.656
Class I or III AADs on discharge	26.0% (539)	25.6% (155)	24.8% (121)	26.4% (115)	33.1% (48)	0.210
Class I AADs on discharge	7.6% (189)	9.7% (69)	7.1% (77)	5.3% (27)	8.6% (16)	0.029
Class III AADs on discharge	14.0% (350)	12.1% (86)	13.2% (144)	17.3% (88)	17.2% (32)	0.030

Values are given as mean±SD or number and (%). AAD indicates anti‐arrhythmic drugs; AF, atrial fibrillation; CFAE, complex atrial fractionated electrogram; CHA_2_DS_2_‐VASc, cardiac failure or dysfunction, hypertension, age ≥75 years [doubled], diabetes mellitus, stroke [doubled]‐vascular disease, age 65 to 74 years, sex category [female]; CTI, cavotricuspid Isthmus; eGFR, estimated glomerular filtration rate; LA, left atrium; LVEF, left ventricular ejection fraction; TIA, transitory ischemic attack.

a Mean time to repeat procedure 15±16 months.

b Roof line in 164 patients, mitral isthmus in 38, roof line and mitral isthmus in 329 patients, and additional linear lesions in 41 patients.

### Baseline Differences Across BMI Classes

A higher prevalence of women (more than a third) was observed in the normal weight and morbidly obese groups. The prevalence of non‐paroxysmal forms of AF increased alongside with BMI, accounting for a third of all normal weight individuals and nearly half of overweight patients (*P*<0.001).

The prevalence of risk factors for coronary and cerebrovascular disease, like hypertension, and diabetes mellitus was progressively higher in pre‐obese, obese and morbidly obese patients (all *P*<0.001). Similarly, the prevalence of OSA rose progressively within the different BMI classes (*P*<0.001). CHA_2_DS_2_‐VASc score was slightly higher in obese and morbidly obese patients.

### Safety Outcomes

The incidence of peri‐procedural complications was similar among the 3 patient groups (normal weight 7.0% vs. pre‐obese 5.9% vs. obese 5.3 vs. morbidly obese 5.9; *P*=0.680) (Table 2).

**Table 2:** Efficacy and Safety End Points

Variable	Total Sample (n=2497)	Normal Weight (n=711)	Pre‐Obese (n=1092)	Obese (n=508)	Morbidly Obese (n=186)	Overall *P* Value
Efficacy						
Pulmonary vein isolation	99.0% (2473)	99.3% (706)	98.9% (1080)	99.0% (503)	98.9% (184)	0.863
Relapse during blanking	25.0% (504)	24.1% (141)	22.6% (194)	28.1% (119)	34.7% (50)	0.007
Relapse during first 12 mo	38.1% (916)	35.2% (241)	35.7% (374)	43.6% (216)	48.0% (85)	<0.001
Safety						
Per‐procedural complications	6.1% (151)	7.0% (49)	5.9% (64)	5.3% (27)	5.9% (11)	0.680
Cardiac tamponade	0.7% (18)	0.3% (2)	0.9% (10)	0.8% (4)	1.1% (2)	0.414
TIA	0.1% (3)	0.1% (1)	0.2% (2)	0% (0)	0% (0)	0.748
Stroke	0.2% (6)	0.1% (1)	0.3% (3)	0.4% (2)	0% (0)	0.730
Transient phrenic nerve palsy	1.5% (37)	1.5% (11)	1.5% (16)	0.8% (4)	3.2% (6)	0.134
Major vascular complications	2.6% (65)	3.5% (25)	2.5% (27)	2.4% (12)	0.5% (1)	0.130
Procedure‐related death^a^	0.1% (1)	0% (0)	0.1% (1)	0% (0)	0% (0)	0.732
Other complications^b^	0.8% (21)	1.3% (9)	0.5% (5)	1.0% (5)	1.1% (2)	0.295
Other complications						
Esophageal fistula	0.1% (2)	0% (0)	0% (0)	0.2% (1)	0.5% (1)	0.067
Gastroparesis	0.1% (2)	0.1% (1)	0% (0)	0.1% (1)	0% (0)	0.529
Esophageal ulcer	0.1% (1)	0.1% (1)	0% (0)	0% (0)	0% (0)	0.473
Non‐access related bleeds	0.2% (6)	0.6% (4)	0.1% (1)	0.1% (1)	0% (0)	0.206
Bradyarrhythmic complications	0.2% (5)	0.1% (1)	0.3% (3)	0.2% (1)	0% (0)	0.849
Anaphylaxis	0.1% (1)	0%(0)	0.1% (1)	0% (0)	0% (0)	0.732
Transient myocardial stunning	0.1% (1)	0% (0)	0% (0)	0.2% (1)	0% (0)	0.271
PV stenosis	0.1% (1)	0.1% (1)	0% (0)	0% (0)	0% (0)	0.473
Air embolism	0.1% (1)	0.1% (1)	0% (0)	0% (0)	0% (0)	0.473
Acute pulmonary edema	0.1% (1)	0% (0)	0% (0)	0% (0)	0.5% (1)	0.006

Values are given as number and (%), and incidence and (95% CI). TIA indicates transient ischemic attack.

a Death occurred as a result of diffuse lung bleed without identifiable source.

b Other complications are as follows: normal weight patients: haemothorax and haemomediastinum (n=1), upper gastrointestinal bleed (n=1), haematuria (n=1), haemoptysis (n=1), gastroparesis (n=1), esophageal ulcer (n=1), and complete atrioventricular block (n=1), PV stenosis (n=1), and air embolism into coronary artery (n=1); Pre‐obese patients: upper gastrointestinal bleed (n=1), sinus node dysfunction requiring permanent pacemaker implant (n=2), reversible period of complete atrioventricular block (n=1), and anaphylactic shock (n=1); Obese: haemoptysis (n=1), sinus node dysfunction requiring permanent pacemaker implant (n=1), esophageal fistula (n=1), temporary myocardial stunning with transient drop in left ventricular ejection fraction (n=1), and gastroparesis (n=1); Mordibly obese: esophageal fistula (n=1), and acute pulmonary edema (n=1). PV indicates pulmonary vein.

The incidence of cardiac tamponade, other bleeds, major vascular complications, transient phrenic nerve palsy, and stroke, transient ischemic attack, or systemic embolism was low and comparable.

The only 2 patients diagnosed with atrio‐esophageal fistula belonged to the 2 higher BMI patient groups (*P*=0.067). One patient in the morbid obesity group develop acute pulmonary edema during the ablation (*P*=0.006). The incidence of specific types of complications was similar across the different BMI groups (Table 2). All but 2 cases of phrenic nerve palsy reverted within the first month. Among the 42 patients with a BMI≥40 kg/m^2^ only 1 complication was reported (cardiac tamponade in a male patient with a BMI of 35.6 kg/m^2^). On multivariate analysis, age and use of NOACs were the only independent predictors of occurrence of complications (Table S1). BMI was not associated with any of the reported complication types.

Nine patients (0.4%) died during follow‐up and no significant differences were observed in mortality rate across the different categories (normal weight =3; pre‐obese =4; obese=2; morbidly obese=0; *P*=0.858). A pre‐obese patient who underwent redo radiofrequency ablation for persistent AF died 15 days following the procedure, as the result of haemoptysis, which started in the same day of the procedure. All other deaths occurred following the blanking period and were classified as non‐procedure related. Cardiac causes were observed in 3 patients: A normal weight and a pre‐obese patient died suddenly at 8 and 19 months, and a pre‐obese individual died of heart failure in the first year; a pre‐obese patient died with opportunistic infection in the setting of HIV after 12 months; the 2 obese patients died of infectious causes in the first year (endocarditis and pneumonia). The remaining 2 patients died of cancer‐related causes (both were normal weight).

### Efficacy Outcomes

Relapse during blanking was significantly more frequent in patients with BMI >35 kg/m^2^.

>At 12 months, 38.1% of patients presented with documented atrial arrhythmia relapse. The rate of relapse at 12 months increased progressively across the 4 BMI classes: 35.2% in normal weight individuals, 35.7% in pre‐obese, 43.6% in obese patients, and 48.0% in morbidly obese patients (*P*<0.001).

**Figure 1:**
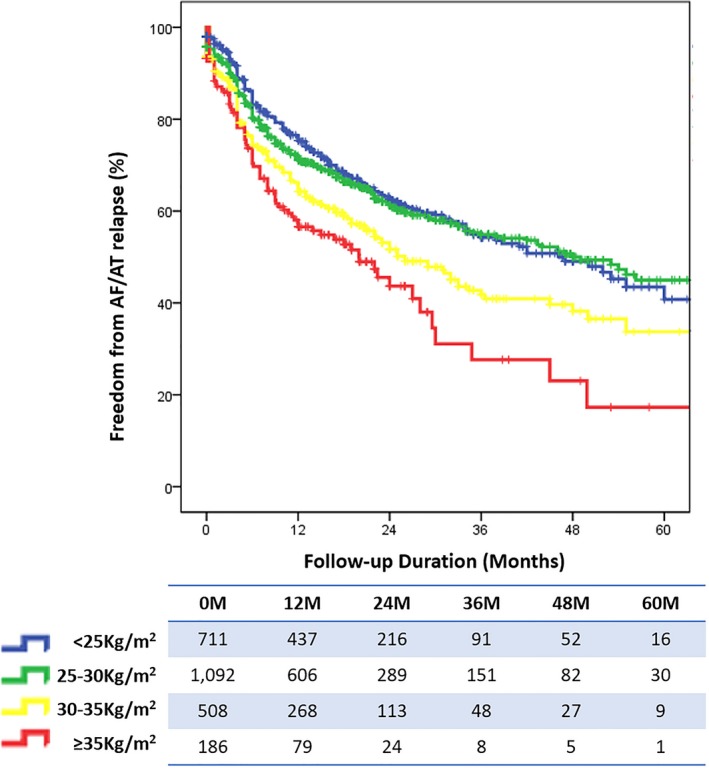
Freedom from atrial arrhythmia relapse stratified by BMI class for all AF patients. AF indicates atrial fibrillation; AT, atrial tachycardia; BMI, body mass index.

During a median follow‐up of 18.8 months (interquartile range 11–28) atrial arrhythmia relapse was higher in the obese patients’ group (log rank *P*<0.001). Despite the initial divergence of all 4 curves, from 12 months onward, curves illustrating freedom from arrhythmia relapse in normal weight and pre‐obese individuals overlapped (Figure 1). Patients in higher BMI categories experienced higher relapse rates.

**Figure 2:**
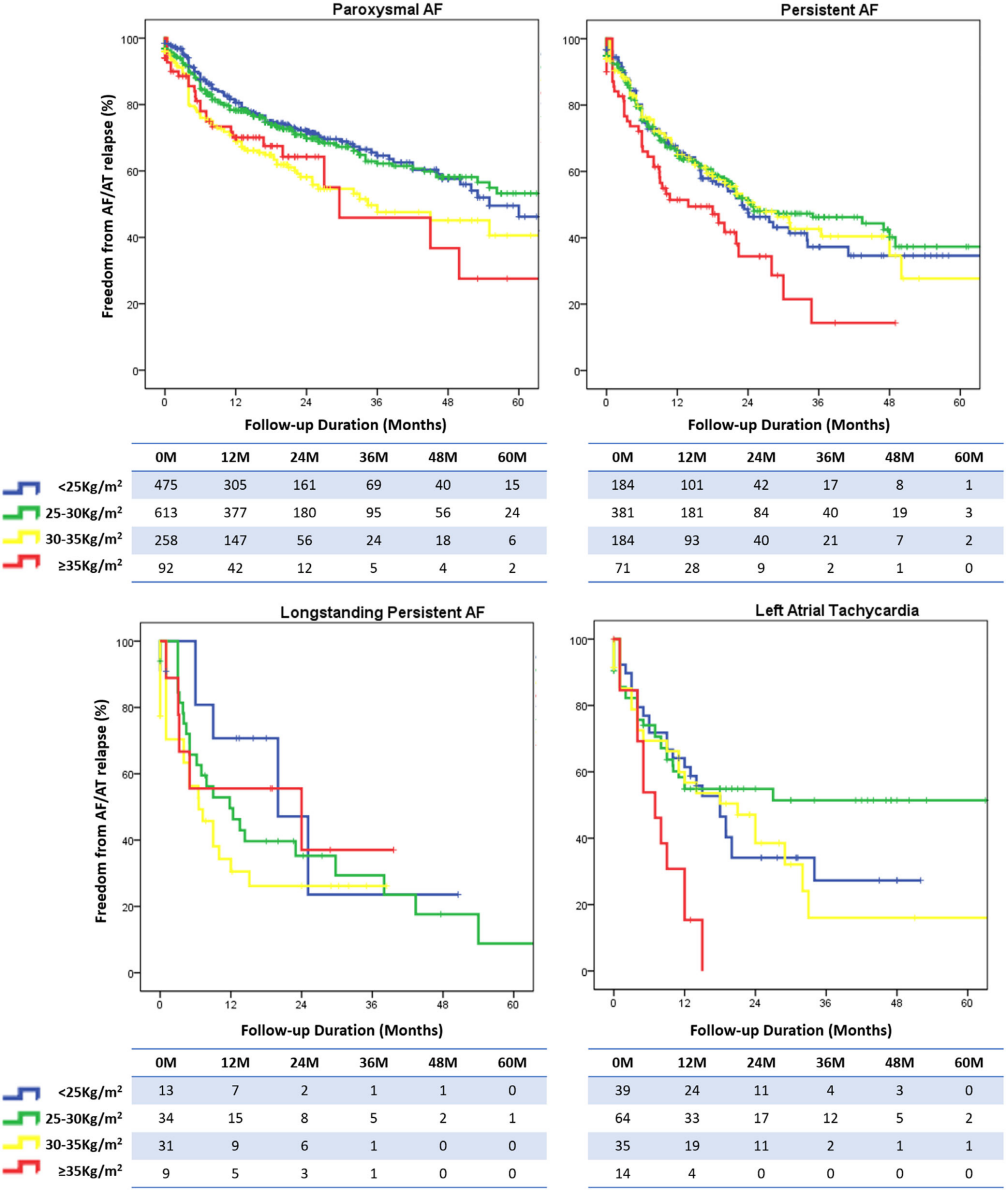
Freedom from atrial arrhythmia relapse stratified by BMI class and AF type. AF indicates atrial fibrillation; AT, atrial tachycardia; BMI, body mass index.

Figure 2 illustrates follow‐up stratified by BMI class and AF type. Patients with morbid obesity do significantly worse than the other groups and relapse more frequently (log rank *P*<0.001). Obese patients relapse more frequently following paroxysmal AF ablation, but have comparable outcomes for persistent AF.

Assessment of independent predictors of AF or arrhythmia relapse is illustrated in Table 3. On multivariate Cox regression, total AF duration in years, paroxysmal AF, diabetes mellitus, BMI, and obstructive sleep apnea, and annual case load were independent predictors of relapse.

**Table 3:** Predictors of Post‐Blanking Atrial Arrhythmia Relapse After an Ablation Procedure

Variable	Univariate Cox Regression			Multivariate Cox Regression—1			Multivariate Cox Regression—2		
	HR	95% CI	*P* Value	HR	95% CI	*P* Value	HR	95% CI	*P* Value
Age (per y)	1.01	1.00 to 1.02	0.008	…	…	…	1.00	0.99 to 1.01	0.441
Female sex	1.20	1.02 to 1.41	0.024	…	…	…	1.07	0.89 to 1.29	0.454
AF duration (per y)	1.03	1.02 to 1.04	<0.001	1.03	1.02 to 1.04	<0.001	1.02	1.01 to 1.03	0.001
Paroxysmal AF	0.52	0.45 to 0.60	<0.001	0.53	0.46 to 0.61	<0.001	0.54	0.45 to 0.66	<0.001
Congestive heart failure	1.58	1.24 to 2.00	<0.001	…	…	…	…	…	…
Hypertension	1.23	1.06 to 1.43	0.007	…	…	…	…	…	…
Diabetes mellitus	1.44	1.14 to 1.81	0.002	1.33	1.05 to 1.67	0.016	1.36	1.03 to 1.78	0.027
Stroke or TIA	1.31	1.00 to 1.61	0.051	…	…	…	…	…	…
Vascular disease	1.19	0.94 to 1.53	0.155	…	…	…	…	…	…
Obstructive sleep apnea	1.48	1.15 to 1.91	0.003	1.32	1.03 to 1.69	0.028	1.21	0.92 to 1.58	0.176
CHA_2_DS_2_‐VASc	1.13	1.07 to 1.19	<0.001	…	…	…	…	…	…
BMI (per kg/m^2^)	1.01	1.00 to 1.01	0.044	1.01	1.00 to 1.02	0.017	1.03	1.01 to 1.05	0.001
eGFR (per mL/min)	0.99	0.99 to 1.00	0.073	…	…	…	…	…	…
Indexed LA volume (per mL/m^2^)	1.01	1.01 to 1.02	<0.001	…	…	…	1.01	1.00 to 1.01	0.002
LVEF (per %)	0.99	0.98 to 0.99	0.002	…	…	…	1.00	0.99 to 1.01	0.773
Cryoballoon ablation	0.92	0.78 to 1.08	0.293	…	…	…	…	…	…
Class I or III ADDs on discharge	1.59	1.37 to 1.85	<0.001	…	…	…	1.24	1.02 to 1.51	0.030
Center case‐load (per 100 ablations/y)	0.94	0.91 to 0.97	<0.001	0.94	0.91 to 0.97	<0.001	1.28	1.09 to 1.50	0.003

Model 2 was created using the previously identified predictors, with additional adjustment for age, sex, left ventricular ejection fraction, left atrium volume, and use of anti‐arrhythmic drugs on discharge. AAD indicates anti‐arrhythmic drugs; AF, atrial fibrillation; BMI, body mass index; CHA_2_DS_2_‐VASc, cardiac failure or dysfunction, hypertension, age ≥75 years [doubled], diabetes mellitus, stroke [doubled]‐vascular disease, age 65 to 74 years, sex category [female]; HR, hazard ratio; LA, left atrium; LVEF, left ventricular ejection fraction; TIA, transitory ischemic attack.

Patients with BMI ≥40 kg/m^2^ relapsed with similar rates to their 35 to 39.9 kg/m^2^ counterparts.

### Sub‐Analyses: NOACs and Cryoballoon Ablation in Obese Patients

The different analysis in different weight and BMI categories does not confirm the concerns of potential underdosing of NOACs in obese patients. In fact, no thromboembolic events were observed in this patient group in patients treated with the NOACs. With regards to bleeding complications, the results were comparable with vitamin‐K antagonists (Table 4).

**Table 4:** Sub‐Analyses for Anticoagulation and Ablation Energy in Obese Patients

	RF (n=502)	Cryoballoon (n=190)	*P* Value
Overweight patients (BMI >30 kg/m^2^)			
All complications	4.6% (23)	7.9% (15)	0.090
Cardiac tamponade	1.0% (5)	0.5% (1)	0.552
Stroke	0.2% (1)	0.5% (1)	0.474
Phrenic nerve palsy	0.4% (2)	4.2% (8)	<0.001
Vascular complications	1.6% (8)	2.6% (5)	0.369
Other bleeds	0.2% (1)	0% (0)	0.538
Bradyarrhythmic complications	0.2% (1)	0% (0)	0.538
Gastroparesis	0.2% (1)	0% (0)	0.538
Transient myocardial stunning	0.2% (1)	0% (0)	0.538
Acute pulmonary edema	0.2% (1)	0% (0)	0.538

	NOAC (n=180)	VKA (n=512)	*P* Value
Overweight patients (BMI >30 kg/m^2^)			
Stroke	0% (0)	0.4% (2)	0.401
TIA	0% (0)	0% (0)	N.A.
Systemic embolism	0% (0)	0% (0)	N.A.
Cardiac tamponade	0.6% (1)	1.0% (5)	0.600
Vascular complications	1.7% (3)	2.0% (10)	0.808
Other bleeds	0% (0)	0.2% (1)	0.538

	NOAC (n=61)	VKA (n=123)	*P* Value
Patients with BMI >35 kg/m^2^			
Stroke	0% (0)	0% (0)	N.A.
TIA	0% (0)	0% (0)	N.A.
Systemic embolism	0% (0)	0% (0)	N.A.
Cardiac tamponade	0% (0)	1.6% (2)	0.317
Vascular complications	1.6% (1)	0% (0)	0.154
Other bleeds	0% (0)	0% (0)	N.A.

	NOAC (n=13)	VKA (n=26)	*P* Value
Patients with BMI >40 kg/m^2^			
Stroke	0% (0)	0% (0)	N.A.
TIA	0% (0)	0% (0)	N.A.
Systemic embolism	0% (0)	0% (0)	N.A.
Cardiac tamponade	0% (0)	3.8% (1)	0.474
Vascular complications	0% (0)	0% (0)	N.A.
Other bleeds	0% (0)	0% (0)	N.A.

	NOAC (n=23)	VKA (n=36)	*P* Value
Patients weighting >120 kg			
Stroke	0% (0)	0% (0)	N.A.
TIA	0% (0)	0% (0)	N.A.
Systemic embolism	0% (0)	0% (0)	N.A.
Cardiac tamponade	0% (0)	0% (0)	N.A.
Vascular complications	0% (0)	0% (0)	N.A.
Other bleeds	0% (0)	0% (0)	N.A.

BMI indicates body mass index; NOAC, novel oral anticoagulants; RF, radio frequency; TIA, transient ischemic attack; VKA, vitamin K antagonists.

Cryoballoon ablation procedures in obese patients were of shorter duration and required slightly lower duration of fluoroscopy screening (radio frequency [RF]: 144±60 min vs. cryoballoon ablation [Cryo]: 115±44 min, and 24±14 min vs. 22±10 min, both *P*<0.001). However, after removing patients receiving additional left atrial and right atrial substrate ablation (in the RF group 134 patients received complex atrial fractionated electrogram ablation, 206 had left atrial linear ablation, and 41 underwent cavotricuspid isthmus ablation; only 17 patients in the Cryo group received cavotricuspid isthmus ablation), comparison of patients receiving only pulmonary vein isolation showed no significant differences in fluoroscopy screening duration (dropping to 23±9 min in the RF group; *P*=0.225). Procedural time in the RF group remained significantly longer, 20 minutes in average (135±39 min; *P*=0.004).

**Figure 3:**
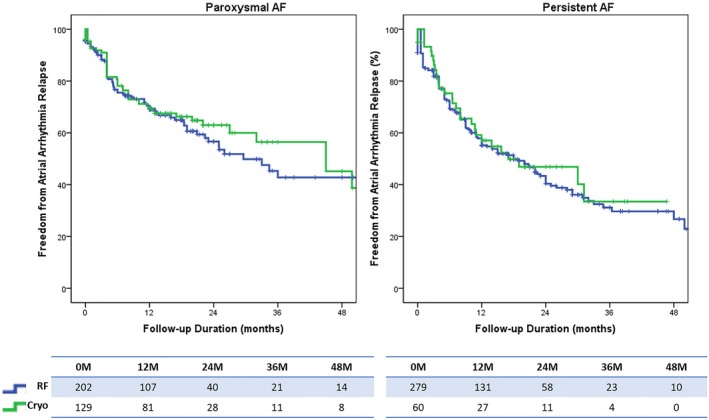
Sub‐analysis on cryoballoon vs radiofrequency ablation in patient with BMI >30 kg/m^2^. Log rank *P*=0.561 for paroxysmal atrial fibrillation and log rank *P*=0.721 for persistent AF. AF indicates atrial fibrillation; BMI, body mass index.

Use of general anesthetic was slightly higher in obese patients undergoing cryoballoon ablation (83.2%, n=158 vs. 63.9%, n=321; *P*<0.001). With regards to efficacy (Figure 3) and safety (Table 4), cryoballoon ablation was comparable with radiofrequency ablation in this patient group.

## Discussion

Our data show that patients with higher BMI undergoing catheter ablation of AF have a higher prevalence of comorbidities‐hypertension, diabetes mellitus, and OSA, and more frequently present with non‐paroxysmal AF forms. Furthermore, obese patients have a higher relapse rate of atrial arrhythmias during follow‐up. After adjustment for confounding factors like cardiovascular risk factors and OSA, on multivariate Cox regression, BMI remained an independent predictor of relapse. On the other hand, BMI was not a predictor of complications. Finally, our data show comparable performance and safety of cryoballoon ablation and NOACs in this patient population.

Some of the previous observational studies^16^, ^17^ failed to show a higher relapse rate in obese patients undergoing catheter ablation of AF. However, these studies were composed of much smaller samples. However, 2 recent publications of single‐center data support our findings: *Winkle* and colleagues in a cohort of 2715 patients demonstrated an association with higher BMI values and atrial arrhythmia relapse.^18^ Unlike what we observed in our data, which constitutes the largest multicenter cohort, in these authors’ cohort, a higher relapse rate in paroxysmal AF was only observed for patients with BMI ≥40 kg/m^2^. Also, we could not confirm the higher risk of complications suggested for that BMI class. In Sivasambu et al's cohort of 701 patients, a higher relapse rate in higher BMI classes was only observed for paroxysmal AF.^19^

We performed extensive adjustment for confounding variables in obese patients, namely OSA and other comorbidities that are highly prevalent in obese patients, and BMI remained an independent predictor of relapse. As previously suggested by Mohanty and colleagues about metabolic syndrome and OSA,^20^ in our cohort we have observed that BMI and obstructive sleep apnea independently increase arrhythmia recurrence following AF ablation.

Our analysis by AF type shows that high BMI is associated with lower procedural success both in paroxysmal AF, and persistent AF. Furthermore, BMI remained an independent predictor of atrial arrhythmia recurrence even after adjustment for AF type, suggesting that response to catheter ablation is worse not only because these patients present with more advanced forms of AF, but also because of obesity‐related factors.

It has been suggested that increased pericardial fat could lead to higher recurrence rate following catheter ablation of AF.^21^, ^22^ A harmful paracrine/vasocrine effect as well as a mechanical action of epicardial fat on myocardium are among the proposed mechanisms underlying this association.^23^ This can happen has a result of the release of circulating adipokines triggering the inflammatory cascade,^24^ or by the compression of the heart by epicardial fat, impairing diastolic filling and causing left ventricular hypertrophy and left atrial dilation.^21^, ^25^ However, we did not observe any differences in indexed left atrial size in our sample across the 4 different BMI categories.

BMI is associated with a higher risk of new‐onset AF.^26^, ^27^ Mahajan et al recently showed in an animal model that sustained obesity leads to global biatrial endocardial remodeling characterized by left atrial enlargement, conduction abnormalities, fractionated electrograms, increased profibrotic transforming growth factor Beta (TGF‐β_1_) expression, and interstitial atrial fibrosis.^28^ Therefore, we wonder that following a successful ablation with pulmonary vein isolation (PVI), if weight reduction is not pursued, obesity may act as a trigger for the development of arrhythmia relapse, by promoting the development of additional extra‐pulmonary vein foci as a result of ongoing and progressive deleterious electroanatomical atrial remodeling.

Our findings may suggest a potential benefit of weight reduction in these patients to counteract the deleterious effect of high BMI. This requires testing a prospective randomized trial, but to date results of the ARREST‐AF (Aggressive Risk Factor Reduction Study for Atrial Fibrillation and Implications for the Outcome of Ablation)^29^ study focusing on aggressive risk factor reduction in individuals with a BMI ≥27 kg/m2 and at least 1 risk factor (hypertension, glucose intolerance/diabetes mellitus, hyperlipidemia, OSA, smoking, or alcohol excess) have shown that this seems to improve the long‐term success of AF ablation. If we consider that weight reduction by itself can lead to improvements in OSA, hypertension, and diabetes mellitus, this provides more support for its potential role in optimizing outcomes of patients who had AF ablation. In a multicenter observational study, metabolic syndrome and OSA were shown to increase arrhythmia recurrence following ablation.^20^ Among patients with relapse, those assigned to aggressive lifestyle modification in addition to previously ineffective anti‐arrhythmic drugs, remained free from arrhythmia relapse in the same proportion as those undergoing a repeat procedure (76% vs. 74%, respectively; *P*=0.71).

In the LEGACY study (Long‐Term Effect of Goal directed weight management on Atrial Fibrillation Cohort: A 5‐Year follow‐up study), sustained weight loss led to a significant reduction of AF burden, and was associated to a beneficial structural remodeling.^30^ These observational data indicate an important reversible component to the pathophysiology of the BMI/AF association, and suggests a crucial role for weight reduction both before and after catheter ablation of AF to optimize the chances of success.

Data from the same group in the CARDIO‐FIT (Impact of Cardiorespiratory Fitness on Arrhythmia Recurrence in Obese Individuals With Atrial Fibrillation) study, also support an additive effect of cardiorespiratory fitness over weight loss.^31^ The ongoing ISOLATE (Impact of Life‐Style Modification On Ablation Outcome in Atrial Fibrillation) study^32^ may shed more light on this subject.

Our results suggest no influence of BMI on safety outcomes. There seemed to be a trend for higher incidence of atrio‐esophageal fistula in obese individuals, but this needs to be confirmed. With regards to acute pulmonary edema, which occurred in 1 morbidly obese patient, or any of the observed rare complications, numbers are too small for strong inferences.

Endoscopic studies have shown that esophageal lesions happen more often in individuals with a low or low‐normal BMI.^33^, ^34^ This is thought to happen because normal weight and underweight individuals have thinner fat pads, and thus a shorter distance between the esophagus and posterior left atrial wall.^35^

Another factor to consider is left atrial dilation, as it is thought to cause “sandwiching” of the esophagus between the left atrium and thoracic spine, and associates with thinner fat pads.^36^ Because both our patients with esophageal fistula underwent extensive ablation (pulmonary vein isolation followed by complex fractionated electrogram ablation and roof line and mitral isthmus ablation) for persistent AF and their atria were dilated (49 and 82 mL/m^2^), we believe that other factors besides BMI may have played a role. Interestingly, Winkle and colleagues reported one fistula in their cohort, and this occurred in the 25 to 30 kg/m^2^ BMI patient group.^18^

Our sub‐analyses on the use of cryoballoon ablation and NOACs in obese patients address a gap in the available evidence for catheter ablation of AF, and appear to suggest the safety of both therapies in this subset of patients. Our data suggest a potential reduction in procedural duration (±20 minutes in average), but no relevant differences with regards to duration of fluoroscopy screening. Furthermore, we acknowledge that since the year 2015 practice in these centers has evolved and screening times are now <10 minutes on average, and for a few operators performing RF procedures, screening times may sometimes be as low as 1 to 2 minutes. For that reason, we believe that data on fluoroscopy screening duration may no longer reflect current practice in mid‐to‐high volume centers.

We acknowledge several limitations in our work. First, this is a multicenter study including experienced centers performing several hundreds of cases annually, and may not represent the type of ablation activity performed in other centers with lower caseloads. Second, use of cryoballoon ablation or NOACs was left at the operator discretion. Even though our data on these therapies in obese patients do not come from a randomized controlled trial, they constitute to the largest available volume of evidence at the moment in this population, and it appears unlikely that randomized controlled trials will ever be run to address these 2 matters. However, results need to be interpreted with some reservation as clinicians chose which patients received which therapy (NOACs and the cryoballoon), and as such, there may be unaccounted selection bias in these analyses. Third, complication rate was low in the obese population, with all different complication types occurring in <1% to 2% of patients. As there were only 180 obese patients on NOACs, the sample is underpowered to show any significant differences in complications versus vitamin‐K antagonists even before adjustment. Interestingly, for a 50% relative reduction in end points occurring in 2% of patients, 2318 patients would be required in each treatment arm (assuming an alpha of 0.05 and 80% power). Sample sizes of the largest trials on comparing NOACs versus Warfarin for AF ablation are clearly smaller than those numbers.^37^ Fourth, no formal assessments of quality of life data were performed in this population, and hence, no comparison of quality of life changes post‐ablation across the different BMI categories was possible. Lastly, comparison of AF burden post‐ablation across the different BMI groups would have been of interest. One‐ to four‐week auto‐triggered ECG monitors were not used in this sample, and only a small minority of our patients (<2%) had been implanted with cardiovascular electronic implantable devices, or implantable cardiac monitors, and hence our numbers were too small for any meaningful comparisons across BMI groups.

## Conclusions

Our findings show that, irrespective of associated comorbidities, a higher BMI is associated with lower success rate in terms of freedom from atrial arrhythmia over long‐term follow‐up. This effect was seen for paroxysmal and persistent AF in patients with BMI of >35 kg/m^2^. Systematic measures leading to a reduction in BMI should play an important role before, and after the procedure. Use of NOACs and cryoballoon ablation appears to be safe and have comparable efficacy to standard therapeutic options in obese patients. Therefore, NOACs and cryoballoon ablation may be considered a possible option in this patient group.

## Author Contributions

Dr Providencia, and Dr Boveda wrote the first version of the manuscript. Drs Albenque, Chen, Combes, Adragao, Asmundis, Chierchia, Carmo, Schmidt, Defaye, Anselme, Cavaco, and Hunter were involved in the design, and organization. Drs Creta, Honarbakhsh, Combes, Carmo, Sousa, and Jebberi were involved in the data collection and final assembly of the registry database. Dr Providencia performed the statistical analysis. Drs Providencia, Lambiase, and Boveda improved the first versions of the manuscript, which were then submitted to all the remaining authors, who suggested the necessary changes for its improvement. All authors critically revised the paper and collaborated in the elaboration of the final version. The final version of the manuscript was prepared and revised by all authors before the final approval and submission of the manuscript.

## Sources of Funding

Dr Lambiase is supported by University College London Hospitals Biomedicine National Institute of Health Research funding.

## Disclosures

Dr Albenque has received consultant fees from St. Jude Medical and Biosense Webster; Dr Anselme has received compensatory fees from Boston Scientific, Medtronic, and LivaNova; Dr Boveda has received consulting fees from Medtronic, Boston Scientific, and Sorin Group. Asmundis received compensation for teaching purposes and proctoring from AF solutions, Medtronic, Abbott, Biotronik, Atricure and research grants on behalf of the center from Biotronik, Medtronic, St Jude Medical Abbot, Livanova, Boston Scientific Biosense Webster. Chierchia received compensation for teaching purposes and proctoring from AF solutions Medtronic and Biotronik. The remaining authors have no disclosures to report.

## Supporting information


**Table S1.** Predictors of Procedural Complications
