# Supplementary material for: Impact of Body Mass Index on the Outcomes of Catheter Ablation of Atrial Fibrillation: A European Observational Multicenter Study
Source: J Am Heart Assoc. 2019 Oct 4;8(20):e012253. doi: 10.1161/JAHA.119.012253 (PMC6818047; doi:10.1161/JAHA.119.012253)
Supplement: Supplementary file 1 — Table S1. Predictors of Procedural Complications [file JAH3-8-e012253-s001.pdf]

Supplemental Material

Table S1. Predictors of Procedural Complications.

Variable	Univariate Logistic Regression			Multivariate Logistic Regression		
	OR	95%CI	P	OR	95%CI	P
**Age (per year)**	**1.02**	**1.00-1.04**	**0.012**	**1.02**	**1.01-1.04**	**0.007**
**Female sex**	1.20	0.84-1.70	0.318	-	-	-
**AF duration (per year)**	1.02	0.99-1.01	0.102	-	-	-
**Paroxysmal AF**	0.91	0.66-1.27	0.591	-	-	-
**Congestive heart failure**	1.17	0.66-2.07	0.583	-	-	-
**Hypertension**	0.81	0.58-1.13	0.205	-	-	-
**Diabetes mellitus**	0.59	0.3-1.17	0.133	-	-	-
**Stroke or TIA**	1.36	0.78-2.37	0.276	-	-	-
**Vascular disease**	0.91	0.49-1.66	0.748	-	-	-
**Obstructive Sleep apnea**	1.24	0.69-2.24	0.475	-	-	-
**CHA_2DS_2-VASc**	1.06	0.94-1.19	0.351	-	-	-
**BMI (per Kg/m^2)**	0.98	0.94-1.01	0.187	-	-	-
**eGFR (per ml/min)**	0.99	0.98-0.99	0.040	-	-	-
**Indexed LA volume (per mL/m^2)**	0.99	0.98-1.00	0.196	-	-	-
**LVEF (per %)**	1.01	0.99-1.03	0.394	-	-	-
**NOAC**	**0.64**	**0.43-0.97**	**0.033**	**0.61**	**0.40-0.92**	**0.018**
**Center Case-Load (per 100 ablations/year)**	1.10	1.01-1.20	0.030	-	-	-

HR – hazard ratio; CI – confidence interval; AF - atrial fibrillation; TIA - transitory ischemic attack; CHA_2DS_2-VASc - cardiac failure or dysfunction, hypertension, age ≥75 years [doubled], diabetes, stroke [doubled] - vascular disease, age 65–74 years, sex category [female]; BMI - body mass index; LA - left atrium; LVEF - left ventricular ejection fraction.
